# Optimal Constrained Stationary Intervention in Gene Regulatory Networks

**DOI:** 10.1155/2008/620767

**Published:** 2008-04-16

**Authors:** Babak Faryabi, Golnaz Vahedi, Jean-Francois Chamberland, Aniruddha Datta, Edward R Dougherty

**Affiliations:** 1 Department of Electrical and Computer Engineering, Texas A&M University, College Station, TX 77843, USA; 2 Computational Biology Division, Translational Genomics Research Institute, Phoenix, AZ 85004, USA

## Abstract

A key objective of gene network modeling is to develop intervention strategies to alter regulatory dynamics in such a way as to reduce the likelihood of undesirable phenotypes. Optimal stationary intervention policies have been developed for gene regulation in the framework of probabilistic Boolean networks in a number of settings. To mitigate the possibility of detrimental side effects, for instance, in the treatment of cancer, it may be desirable to limit the expected number of treatments beneath some bound. This paper formulates a general constraint approach for optimal therapeutic intervention by suitably adapting the reward function and then applies this formulation to bound the expected number of treatments. A mutated mammalian cell cycle is considered as a case study.

## 1. Introduction

One objective of genetic regulatory modeling is to design intervention strategies that affect the evolution of the gene activity profile of the network. Such strategies can be useful in identifying potential drug targets and treatment methods to alter network evolution in some desirable manner. The states of the network can be partitioned into two sets, desirable and undesirable, which correspond to functional cellular states, such as proliferation and apoptosis [1]. In biology, there are numerous examples where the (in)activation of one gene or protein can lead to a certain cellular functional state or phenotype. For instance, consider a stable cancer cell line borrowed from [2]. Without intervention, the cell cycle continues and cancerous cells proliferate with time. If the goal of the intervention is to push the cells into apoptosis, or programmed cell death, to stop the cell cycle one can use the p gene. The p gene is the most well-known tumor suppressor gene, encoding a protein that regulates the expression of several genes such as Bax and Fas/APO-1, which function to promote apoptosis [3,4]. In cultured cells, extensive experimental results indicate that when p is activated, for example, in response to radiation, it leads to cell growth inhibition or cell death [5]. The p gene is also used in gene therapy, where the target gene (p in this case) is cloned into a viral vector. The modified virus serves as a vehicle to transport the p gene into tumor cells to generate intervention [6,7].

As this and many other examples suggest, it is prudent to use external variables to beneficially alter the evolution of gene regulatory networks. The design of intervention strategies that reduce the likelihood of states favorable to metastasis in cancerous cells has been recently studied by the systems biology community [2,8]. In particular, regulatory intervention has been studied in the context of probabilistic Boolean networks (PBNs) [9]. These networks, which allow the incorporation of uncertainty into the inter-gene relationships, are essentially probabilistic generalizations of the standard Boolean networks introduced by Kauffman [10–12]. In a PBN, gene values are selected from a finite set of quantization levels. The values are updated synchronously at each updating epoch according to regulatory functions. The regulatory functions are allowed to change at time points selected by a binary switching random variable. This incorporates the effect of latent variables outside the model, whose behaviors influence regulation within the model. In essence, the PBN is composed of a collection of networks; between switches it acts like one of the constituent networks. The PBN model also allows random perturbation of genes at each updating instant.

Under appropriate assumptions, a Markov chain models the dynamical behavior of a PBN [9,13]. An optimal intervention strategy is developed based on the associated Markov chain. Methods have been proposed to devise effective intervention strategies. A one-time intervention has been designed based on first-passage times [14]. Dynamic programming can also be used to design optimal finite-horizon control policies [15]. Alternatively, Markov decision processes can be employed to find stationary intervention strategies that alter the steady-state distribution of the state space [16]. Recently, model-free methods have been introduced based on reinforcement learning [17] and mean first-passage times [18] to reduce the likelihood of visiting undesirable states in the long run.

Common to these approaches is a utility function that is to be maximized in order to reduce the aggregated probability of disease states. In reality, treatment options, for example, chemotherapy, cause collateral damages. For instance, consider a second example borrowed from [2]. A treatment based on estrogen is often used by women after menopause to alter their accelerated aging trend. The amount of estrogen received during treatment should not exceed a threshold, since an overdose may increase the chance of developing breast and ovarian cancers. While this phenomenon is not fully understood, it is conceivable that estrogen therapy may have side effects on gene regulation. Estrogen generates two types of complexes through binding to two classes of receptors. The generated complexes are transported into the nucleus to bind to the enhancer elements on the target genes with the help of a coactivator. The coactivator is also required for efficient transcriptional regulation by estrogen. This function in cooperation with a coactivator acts like a transcription factor, affecting target genes such as the PENK gene [19]. Two types of receptors are competing for binding to the estrogen received via treatment [20]. The first type of complex binds DNA better but performs less efficiently to bind the coactivator. On the other hand, the second type of complex binds the coactivator better but performs poorly when binding DNA. When the level of estrogen is below a threshold, there is no competition for DNA binding. Hence, the second type of complex binds DNA and activates the downstream target gene PENK, with the help of its coactivator. However, when the estrogen level is high, both types of complex exist at high concentrations, and the second type of complex binds the coactivator. Consequently, the level of coactivator available to complex type one drops, so the complex type two has a small chance to bind to DNA, and cannot activate the target gene. If the PENK gene plays a role in tumor suppression, for instance, then this could explain why high levels of estrogen have a tumorigenic effect. An appropriate treatment strategy mitigates this problem by bounding the expected number of treatments received by a patient and, as a consequence, limits the dose of estrogen.

Using constrained intervention methods, we seek an effective regulatory treatment that reduces the likelihood of visiting undesirable gene-activity profiles, that is, state, in the long run while providing an upper bound on the expected number of interventions a patient can receive. Instead of introducing a single utility function whose maximization reduces the likelihood of entering undesirable states, we consider a situation where one type of utility is maximized while keeping the other cost function below a given threshold. Posed this way, the intervention problem can be viewed as a constrained Markov decision process.

In our framework, a gene regulatory network is modeled as a dynamical system in which decisions regarding treatment are taken sequentially. We wish to design an intervention strategy that selects treatments (actions) as a function of time and available information. For a given intervention strategy, the choice of treatments at different decision epochs may depend on the whole observed history. The choice of an intervention strategy will determine the evolution of the state of an intervened biological system in some probabilistic sense. The trajectories of the states together with the choice of treatments determine the expected utility in conjunction with the expected cost that we encounter. Hence, the proposed method enables us to design therapeutic intervention strategies by defining problem dependent constraints. Although various forms of constraints are plausible, hereafter, we focus on the expected number of treatments.

We provide the necessary background and formulate the problem of unconstrained intervention in a PBN as a Markov decision process in Section 2. The constrained intervention method is formulated in Section 3. As a numerical study, in Section 4, we consider a network obtained from the mammalian cell cycle with mutated phenotype. We design a constrained intervention strategy to hinder cell growth in the absence of growth factors, while keeping the expected number of interventions bounded. We investigate how the constrained intervention strategy performs in comparison to the unconstrained policy.

## 2. Unconstrained Optimal Intervention in Probabilistic Boolean Networks

A probabilistic Boolean network (PBN) consists of a sequence  of  nodes, where , and a sequence  of vector-valued functions called predictor functions. In the framework of gene regulation, each element  represents the expression value of a gene. It is common to mix the terminology by referring to  as the th gene. Each vector-valued function  determines a constituent network of the PBN. The function  is a predictor of gene , whenever network  is selected. The number of quantization levels is denoted by . At each updating epoch, a decision is made whether to switch the constituent network. The switching probability  is a system parameter. If the network is not switched, then the PBN behaves like a fixed network and synchronously updates the values of all the genes according to the current predictor function. If the network is switched, then a predictor function is randomly selected according to probability distribution . After selecting the predictor function , the values of genes are updated accordingly, that is, according to the network determined by . We consider PBNs with perturbation, in which each gene may change its value with a small perturbation probability  at each time unit.

Two quantization levels have thus far been used in practice. If  (binary), then the constituent networks are Boolean networks with  or  meaning OFF or ON, respectively. The case  (ternary) arises when we consider a gene to be down-regulated (0), up-regulated (2), or invariant (1). This situation commonly occurs with cDNA microarrays, where a ratio is taken between the expression values on the test channel (red) and the base channel (green). In this paper, we will develop the methodology for , so that gene values are either  or ; however, the methodology is applicable to any finite number of levels.

The gene-activity profile (GAP) is an -digit binary vector  giving the expression values of the genes at time , where . We note that there is a natural bijection between the GAP  and its decimal representation, which takes values in .

In the presence of external controls, we suppose that the PBN has  binary control inputs, , which specify the interventions on control genes . A control , which can take values  or  at each updating epoch , specifies the action on the control gene . The decimal bijection of the control vector, , describes the complete status of all the control inputs. As in previous applications, we focus on a single control gene , which we label by , possessing the control function . The treatment alters the status of the control gene , which can be selected among all the genes in the network. If the control at updating epoch  is on, , then the state of the control gene  is toggled; if , then the state of the control gene  remains unchanged.

Brun et al. showed that the dynamic behavior of a PBN can be modeled by a Markov chain [13]. In this case, system evolution for a single control gene  is represented by a stationary discrete-time equation:(1)

where state  is an element of the state-space . The disturbance  is the manifestation of uncertainties in the PBN. It is assumed that both the gene perturbation distribution and the network switching distribution are independent and identical for all time steps . Originating from a state , the successor state  is selected randomly within the set  according to the transition probability :(2)

for all  and  in , and for all  in . Gene perturbation insures that all the states in the Markov chain communicate with one another. Hence, the finite-state Markov chain has a unique steady-state distribution [21].

The problem of optimal intervention for PBNs is formulated as an unconstrained Markov decision process [16]. A reward-per-stage  is associated to each intervention in the system. In general, a reward-per-stage could depend on the origin state , the successor state , and the control input . We assume that the reward-per-stage is stationary and bounded for all ,  in , and  in . We define the average immediate reward in state , when control  is selected, by(3)

We consider the discounted formulation of the expected total reward. The discounting factor, , ensures the convergence of the expected total reward over the long-run [22]. In the case of cancer therapy, the discounting factor emphasizes that obtaining treatment at an earlier stage is favored over later stages. The normalized expected total discounted reward, given policy , initial state , and control gene , is denoted by(4)

A policy  is a sequence of decision rules  for each updating epoch  acting on control gene , given that the initial state is . In general, a decision rule  at updating epoch  selects action  according to the history of the system as well as the current state. The history  at the updating epoch  is composed of the sequence of previous states and actions. If the history  is observed at the updating epoch , then the decision rule  determines the probability of selecting action  conditioned on the history  and the current state . We denote the set of all such policies by , when gene  is selected as the control gene. The set  is the subset of Markovian policies within the set of all policies  defined above. A policy is Markovian if given the current state  the decision rule  is independent of all the previous states and actions , and selects action  with probability  at decision epoch . We denote the set of all stationary policies by , where a stationary policy for control gene  is an admissible intervention strategy in  of the form . Here,  denotes a time invariant decision rule. A stationary policy is also a deterministic policy if decision rule  is deterministic and time invariant for each updating epoch . The set of all deterministic policies is represented by .

Frequently, the discounted reward is defined without the normalizing constant . This constant does not change the method and the solution of the intervention policy. However, using the normalizing constant has several advantages. First, this prevents the total reward from growing excessively for values of  close to one. Second, the use of the normalization constant provides an interesting interpretation for the total cost in the constrained intervention design. This will become clear in the later sections of the paper.

The vector  of normalized expected total discounted rewards is called the value function. In an unconstrained intervention problem, we seek an admissible intervention strategy  that maximizes the value function for each initial state , that is,(5)

It is known that an optimal intervention strategy exists for the unconstrained discounted intervention problems, and it is given by the fixed-point solution of the Bellman optimality equation:(6)

Moreover, an optimal policy determined by the Bellman optimality equation is deterministic, and independent of the initial state [22]. Standard dynamic programming algorithms can be used to find a fixed-point of the Bellman optimality equation.

## 3. Constrained Intervention Inprobabilistic Boolean Networks

Cancer therapy may include the use of chemotherapy, radiation therapy, targeted gene therapy, and so forth. All of these treatment options are directed at killing or eradicating cancerous cells. Unfortunately, cancer treatments may also damage healthy cells. This results in complications and harmful side effects. It is therefore desirable to maintain the side effects of a treatment to a minimum. This goal can be achieved by enforcing an upper bound on the expected number of treatments a patient may receive during therapy. A deterministic intervention policy devised by solving the unconstrained optimization (5) reduces the chance of visiting undesirable states; however, this intervention policy does not provide a way to constrain the frequency of applying treatments within a prescribed intervention policy. To address this shortcoming, we impose an appropriate constraint on the optimization problem (5) by introducing constrained intervention in PBNs.

For the same reasons articulated in Section 2, we consider a discounted formulation to define both the objective reward function and constraint cost function. To restrict the frequency of applying intervention, we associate a cost-per-stage  to each state-action pair  in the constrained formulation. The set of all possible state-action pairs is denoted by . A cost-per-stage should be defined to appropriately reflect the constraint. Here, we bound the discounted expected number of interventions in the long run. Accordingly, the normalized expected total discounted cost of the constraint, given policy , initial state , and control gene  is denoted by(7)

Having the constrained cost function defined this way and the objective reward function as in (4), we can state the constrained intervention problem in a PBN as(8)

where  is the upper bound on the discounted expected number of interventions in the long run, and  is the initial state.

We wish to find an optimal intervention policy  within the set of admissible policies  (not just Markovian policies) that maximizes the value function while satisfying the constraint imposed on the discounted expected total cost. Interventions using policy  increase the time spent in desirable states, while limiting the discounted expected number of treatments. The intervention strategy is determined through the appropriate assignments of reward-per-stage and cost-per-stage to each state-action pair.

Given an arbitrary policy  and starting from initial state , the state trajectories and selected actions over time are probabilistic. Our objective is to find the expectation of the number of times that state-action pairs  with active intervention decision,  occur over the progression of the PBN. This value corresponds to the expected number of treatments in an intervention policy. To this end, we denote the probability that a state-action pair  in the set of all possible state-action pairs  occurs at updating epoch  as(9)

We further define the normalized discounted total expected time spent in the state-action pair  in the long run as(10)

for all , where  is an initial state and  is a policy in . The set(11)

denotes a probability measure over the set of state-action pairs . The numbers of states and actions of a PBN are finite, and the discounting factor  guarantees uniform convergence of (10). The set  for any initial state  and policy  is called an *occupation measure*[23]. The occupation measure can be interpreted as the probability of occupying state-action pairs  in the long run, given that the PBN is initially in state  and policy  is used throughout.

The normalized discounted reward function (4) can be expressed as the expectation of the average immediate reward  over the probability distribution defined in (9):(12)

The normalized discounted reward function in (12) can be equivalently expressed as(13)

Using definition (10) and probability measure (11), we can express the latest form of the normalized discounted reward (13) as the expectation of the average immediate reward with respect to the occupation measure:(14)

Similarly, we can express the normalized discounted objective cost corresponding to policy  as the expectation of the cost-per-stage with respect to the occupation measure:(15)

Using (14) and (15), we can rewrite the constrained optimization problem (8) as(16)

It is evident that the constraint in (16) prevents the discounted expected number of interventions in the long run from exceeding the upper-bound  if we assign the cost-per-stage for each state-action pair in  as(17)

In other words, using the definition of cost-per-stage in (17), the left side of the inequality constraint in (16) corresponds to the total discounted expected number of times that state-action pairs with active treatment, , occur under control policy . Equivalently, we can interpret this as the discounted frequency of applying treatments given a therapeutic strategy.

Several solutions for the constrained optimization problem of (8) are presented in [24]. We next briefly present a method to solve this constrained Markov decision process using the equivalent problem formulation of (16). In [24], it is shown that the set of stationary policies  is complete. In other words, if(18)

denotes the set of all the occupation measures and(19)

denotes the set of occupation measures generated by stationary policies only, then . Further, let  be defined as the set of vectors  that satisfy(20)

where  is indicator function, equaling one if  is true. If , then one can verify that  by summing the first constraint on  in the definition of  over all . Hence, the elements of any  satisfying the constraints in (20) constitute a probability measure on .

It has been shown that , where  and  is the closed convex hull of deterministic policies [24]. Moreover, the closed convex hull of deterministic policies  is equal to the closed polytope specified by . Hence, from the definition in (20) and the cost formulation (15), we can find an optimal policy that satisfies (16) by solving the following linear program:(21)

This linear program is called the primal problem.

In [24], it is shown that an optimal stationary policy  of the constrained optimization problem (16) exists if and only if the primal problem (21) has a solution . Moreover, an optimal solution of (21) uniquely determines an optimal stationary policy . An optimal stationary policy, , thus selects action  at state  with probability:(22)

We should point out that the optimal policy devised by (22) is not necessarily a deterministic policy, in contrast to a policy that maximizes reward function (4) without limitations.

Depending on the utilized numerical method, the computational complexity of finding a solution for the linear program in (21) varies. It is known that the complexity of the interior-point method increases polynomially with the number of states in , where the exponent of the complexity polynomial is not large [25]. Moreover, it is known that the number of iterations required for the numerical method to converge is in the order of , where  is the accuracy of the outcome of the numerical method. Here, the size of  increases exponentially with the number of genes  and the number of controls  in the PBN model with control. The goal, in the application of interest, is not to model fine-grained molecular interactions among a host of genes, but rather to model a limited number of genes, typically with very coarse quantization, whose regulatory activities are significantly related to a particular aspect of a specific disease. Hence, the proposed method is easily up to the task of handling the limited size networks with which we are dealing.

## 4. Constrained Intervention in a Mammalian Cell-Cycle Network

In this section, we construct a PBN that is a probabilistic version of the Boolean model for the mammalian cell cycle regulation proposed in [26]. This PBN postulates the mammalian cell cycle with a mutated phenotype. Our proposed constrained intervention method is then applied with various bounds on the frequency of applying treatments; the therapeutic policy seeks to hinder cell growth in the absence of growth factors.

During the late 1970s and early 1980s, yeast geneticists identified the cell-cycle genes encoding for new classes of molecules, including the cyclins (so-called because of their cyclic pattern of activation) and their cyclin dependent kinases (cdks) partners [26]. Our model is rooted in the work of Fauré et al., who have recently derived and analyzed the Boolean functions of the mammalian cell cycle [26]. The authors have been able to quantitatively reproduce the main known features of the wild-type biological system as well as the consequences of several types of mutations. Mammalian cell division is tightly controlled. In a growing mammal, the cell division should coordinate with the overall growth of the organism. This coordination is controlled via extra-cellular signals. These signals indicate whether a cell should divide or remain in a resting state. The positive signals, or growth factors, instigate the activation of Cyclin D (CycD) in the cell.

The key genes in this model are CycD, retinoblastoma (Rb), and p27. Rb is a tumor-suppressor gene. This gene is expressed in the absence of the cyclins, which inhibit Rb by phosphorylation. Whenever p27 is present, Rb can be expressed even in the presence of CycE or CycA. Gene p27 is active in the absence of the cyclins. Whenever p27 is present, it blocks the action of CycE or CycA. Hence, it stops the cell cycle.

The preceding explanation represents the wild-type cell-cycle model. Following one of the proposed mutations in [26], we assume p27 is mutated and its logical rule is always zero (OFF). In this cancerous scenario, p27 can never be activated. This mutation introduces a situation where both CycD and Rb might be inactive. As a result, in this mutated phenotype, the cell cycles in the absence of any growth factor. In other words, we consider the states in which both Rb and CycD are down-regulated as "undesirable states," when p is mutated. Table 1 summarizes the mutated Boolean functions.

**Table 1:** Mutated boolean functions of mammalian cell cycle.

Product	Predictors
	Input
	
	
	
	
	
	
	
	

The Boolean functions in Table 1 are used to construct the PBN model for the cell cycle. To this end, we assume that the extra-cellular signal to the cell-cycle model is a latent variable. The growth factor is not part of the cell, and its value is determined by the surrounding cells. The expression of CycD changes independently of the cell's content and reflects the state of the growth factor. Depending on the expression status of CycD, we obtain two constituent Boolean networks for the PBN. The first constituent Boolean network is determined from Table 1 when the value of CycD is equal to zero. Similarly, the second constituent Boolean network is determined by setting the variable of CycD to one. To completely define the PBN, the switching probability, the perturbation probability, and the probability of selecting each constituent Boolean network have to be specified. We assume that these are known. Here, we set the switching probability and the perturbation probabilities equal to  and  respectively, and the two constituent Boolean networks are equally likely.

According to Table 1, the mutated cell-cycle's PBN consists of nine genes: CycD, Rb, EF, CycE, CycA, Cdc , Cdh , UbcH , and CycB. The above order of genes is used in the binary representation of the states, with CycD as the most significant bit and CycB as the least significant bit. This order of genes in the states facilitates the presentation of our results and does not affect the computed control policies. Here, the set  denotes the decimal bijection of gene-activity profiles when the above gene order is used for presentation

Preventing the states with simultaneously down-regulated CycD and Rb as our objective, we apply the constrained intervention method described in Section 3 to the constructed PBN with various bounds on the frequency of applying control in a policy. We only consider a single control, . If the control is high, , then the state of the control gene is reversed; if , then the state of the control gene remains unchanged. The control gene can be any of the genes in the model except CycD.

We assume that the reward of the states with down-regulated Rb and CycD is lower than those for the states in which these two genes are not simultaneously down-regulated. We also consider the cost of applying a control action, which reduces the reward of each state. We postulate the following rate-of-reward function:(23)

We select an arbitrary rate of reward; however, the reward and control cost are selected so that applying the control to prevent the undesirable states is preferable in comparison to not applying control and remaining in an undesirable state. In practice, the reward values have to capture the benefits and costs of the intervention and the relative preference of the states. They have to be set in conjunction with physicians according to their clinical judgement. Although this is not feasible within the domain of current medical practice, we do believe that such an approach will become increasingly mainstream once engineering approaches are demonstrated to yield significant benefits in translational medicine. Assuming the preceding rate-of-reward function, we can compute control policies for the PBN associated to the cell-cycle network according to various constraints.

**Figure 1:**
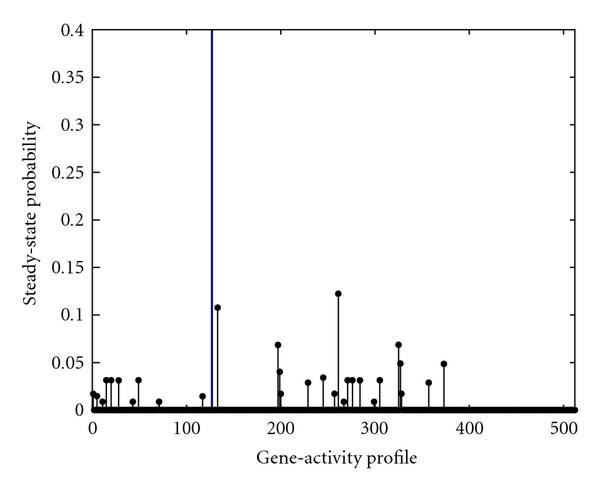
**The steady-state probability of gene-activity profile of the PBN associated with the mammalian cell-cycle network before intervention**. The vertical line separates the undesirable gene-activity profiles from the desirable ones.

Figure 1 depicts the steady-state distribution of the gene-activity profile when there is no intervention. Per Figure 1, in this PBN, the aggregated probability of the gene-activity profiles with simultaneously down-regulated CycD and Rb is close to . In other words, the model predicts that the mutated cell-cycle will be in the cancerous gene-activity profiles  to  nearly  of its time in the long run.

We define  to be the percentage change in the aggregated probability of undesirable gene-activity profiles with simultaneously down-regulated CycD and Rb with and without intervention. As a performance measure,  indicates the percentage of the reduction in the likelihood of cancerous situations in the long run.

If we assume that we can alter the expression level of any gene in the network as a therapeutic method, then it is natural to ask which gene should be used to alter the behavior of the model. To this end, we find a constrained intervention policy for each gene in the network using the intervention method explained in Section 3, while limiting the expected number of times a control can be applied. First, we assume that the PBN's initial state is the undesirable gene-activity profile with the highest probability in the steady-state distribution of gene-activity profiles prior to intervention. Table 2 lists the value of  corresponding to each gene in the network. Here, we vary the upper bound on the frequency of applying intervention and find the corresponding constrained policies.

**Table 2:** The  for the intervention strategy based on various control genes and various constraint bounds.

Control gene										
										
Rb										
EF										
CycE										
CycA										
Cdc										
Cdh										
UbcH										
CycB										

**Figure 2:**
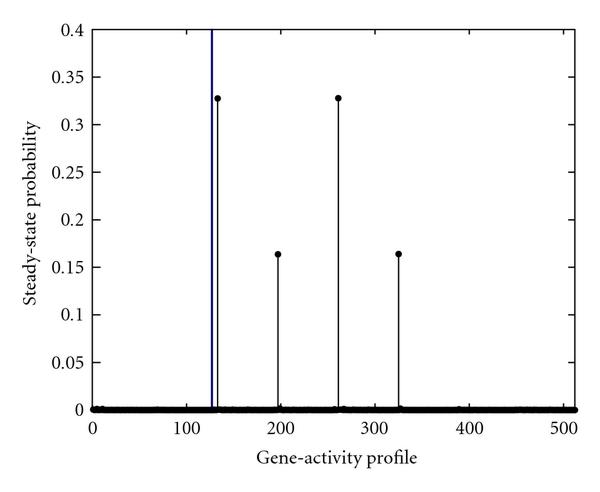
**The steady-state probability of gene-activity profile of the PBN associated with the mammalian cell-cycle network after intervention using Rb as the control gene, when the frequency of applying control is unconstrained, **. The vertical line separates the undesirable gene-activity profiles from the desirable ones.

Among all the genes, Rb offers the best performance when control can be applied without any constraint, based strictly on maximization of the reward function, . After applying the unconstrained control policy designed for Rb, the aggregated probability of undesirable gene-activity profiles is significantly altered (see Figure 2). To avoid the undesirable gene-activity profiles, we utilize the intervention strategy devised by the proposed method in Section 3 for the case when there is no bound on the expected number of treatments. In this scenario, let us assume that the gene-activity profile at a decision epoch indicates that , and . The devised stationary intervention strategy, which is a mapping from the gene-activity profile to the action set , indicates that, for the observed gene-activity profile, the value of control gene Rb should be toggled with probability one. Consequently, we should use an appropriate inhibitor to forcefully down regulate the control gene Rb. Hence, the gene-activity profile would be forced from  to  after this intervention. Although the techniques to implement such a policy, that is, effectively altering the expression of gene Rb, using its enhancers and inhibitors, may not be fully understood within the domain of current medical practice, almost surely these techniques will have detrimental side effects. The constrained stationary intervention designed by the proposed procedure enables us to restrict the expected number of such interventions a patient may receive during therapy. Hence, we could accordingly adjust our intervention strategy when the side effects of drugs effecting the regulation of gene Rb are known.

**Figure 3:**
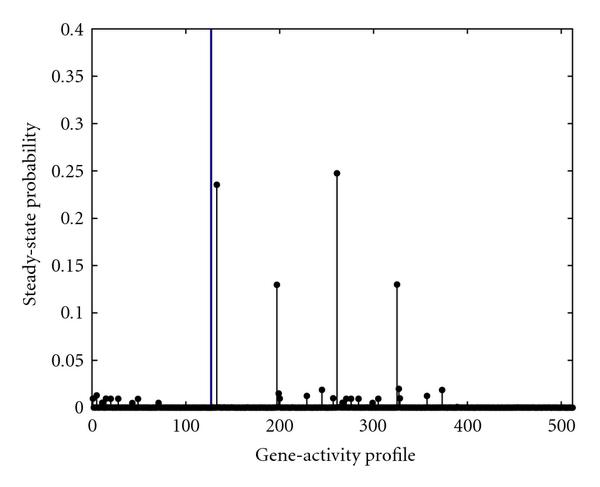
**The steady-state probability of gene-activity profile of the PBN associated with the mammalian cell-cycle network after intervention using Rb as the control gene, when the frequency of applying control is upper bounded by **. The vertical line separates the undesirable gene-activity profiles from the desirable ones.

Figure 3 indicates that by using a constrained stationary intervention policy for the control gene Rb we can reduce the aggregated probability of the undesirable states to less than , while restricting the number of interventions to at most . We could translate this to restrict the dose of prescribed drugs once knowledge of their side effects is available. If we only wish to limit the expected number of applied interventions to less than , then we can reduce the chance of the cancerous gene-activity profiles by .

**Figure 4:**
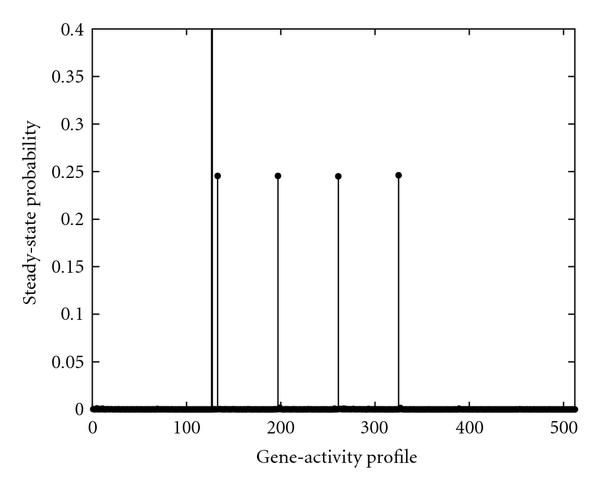
**The steady-state probability of gene-activity profile of the PBN associated with the mammalian cell-cycle network after intervention using EF as the control gene, when the frequency of applying control is is unconstrained, **. The vertical line separates the undesirable gene-activity profiles from the desirable ones.

**Figure 5:**
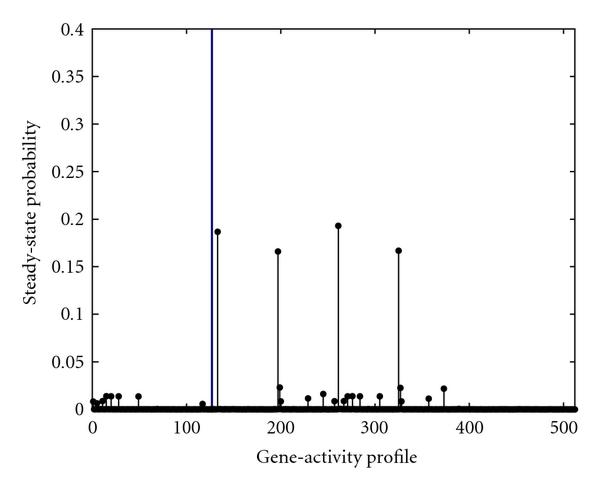
**The steady-state probability of gene-activity profile of the PBN associated with the mammalian cell-cycle network after intervention using EF as the control gene, when the frequency of applying control upper bounded by **. The vertical line separates the undesirable gene-activity profiles from the desirable ones.

According to Table 2, intervention policies based on gene EF performs almost as well as Rb when the constraint is not too tight, . This suggests that, given the side effects of treatments, we may need to consider alternative control genes. The steady-state probability distributions of gene-activity profiles after intervention based on EF are presented in Figures 4 and 5.

Comparing Figures 2 and 4, one can observe that although the final performances of intervening based on these two genes are close, the probability mass of the most probable gene-activity profiles after intervention with Rb differs from the one in EF-based intervention. This observation suggests that one should utilize the systematic analysis along with experimental studies to obtain more effective lever points.

The results of Table 2 indicate that some genes are more sensitive to the bound on the frequency of control. Relaxing the constraint will not improve the result of intervention when the gene UbcH is selected as the control gene. It is simply not an effective lever point. Genes CycB and Cdc perform relatively well for tightly constrained intervention policies but relaxing the limitation on the expected number of treatments does not significantly improve the performance of the policies based on these genes.

Furthermore, if we do not assume that the PBN's initial state is the undesirable gene-activity profile with the highest probability in the steady-state distribution of gene-activity profiles prior to intervention but instead initialize the PBN from an arbitrary undesirable gene-activity profile, we observe that the policies are robust to the initial state unless the constraint is too tight. For , the values of  do not alter significantly; the performance of the intervention policy varies more for different initial gene-activity profiles when the constraint is tight, .

## 5. Conclusion

We have formulated the constrained intervention method in probabilistic Boolean networks and demonstrated that one can reduce the likelihood of a subset of undesirable states while bounding the expected number of interventions in a therapeutic strategy using the proposed method. We have considered a mutated mammalian cell-cycle network in which the cell growth does not stop in the absence of growth factors. We have then utilized the proposed intervention method to design constrained intervention policies to influence the dynamics of the PBN constructed for the mutated mammalian cell cycle. The goal of intervention is to reduce the chance of undesirable cell proliferation in the long run, while maintaining a bound on the expected number of interventions. The presented numerical studies strongly suggest that constrained intervention can effectively alter the dynamics of the cell-cycle model. Various control genes can be considered given different constraints. The most effective control gene may vary depending on the restrictions imposed on the intervention policies.
